# Smart Healthy Age-Friendly Environments – Policy Recommendations of the Thematic Network *SHAFE*

**Published:** 2019-01-06

**Authors:** C Dantas, W van Staalduinen, A Jegundo, J Ganzarain, M Van der Mark, F Rodrigues, M Illario, V De Luca

**Affiliations:** 1 Cáritas Diocesana de Coimbra, Coimbra, Portugal; 2 AFEdemy, Ltd, The Netherlands; 3 Utrecht University, Utrecht, The Netherlands; 4 Health Innovation Division of Campania Region (DG04), Federico II University and Hospital, Naples, Italy; 5 Research and Development Unit, Federico II University Hospital, Naples, Italy

**Keywords:** Joint Statement, SHAFE, eHealth, mHealth, integrated care, age-friendly environments, digital solutions, policy on Health and Care

## Abstract

The European Commission (DG SANTE) launched a call for proposals in November 2017 on strategic initiatives for a Joint Statement in 2018. Ten proposals were voted until December 7th in the European Union Health Policy Platform[1]; the proposal under the theme *Smart Healthy Age-Friendly Environments* (SHAFE)[2] was the most voted and was confirmed by the European Commission in March 2018. In this context, since March 2018, Cáritas Coimbra^1^ and AFEdemy Ltd^2^ are thus coordinating one of the three Thematic Networks for 2018, SHAFE, in close cooperation with main partners, such as the European Innovation Partnership on Active and Healthy Ageing (EIP-AHA), the European Innovation Partnership on Smart Cities and Communities (EIP-SCC), the Reference Sites Collaborative Network, the European Covenant on Demographic Change, Eurocities, the European Health Telematics Association (EHTEL), the European Connected Health Alliance (ECHAlliance), the European Construction, Built Environment and Energy Efficient Building Technology Platform (ECTP) and the European Centre for Social Welfare Policy and Research.

SHAFE aims to facilitate the creation of healthy and friendly environments for all ages through the use of new technologies, towards the production of a comprehensive and participatory Joint Statement. This document was presented to the European Commission on 12 November 2018, with five main areas of recommendations to the EC, Member States and other local, regional and national organisations and is open for endorsement and implementation from this date onwards.

## I. INTRODUCTION

The impact of demographic ageing within the European Union (EU) is likely to be of major significance in the coming decades. Consistently low birth rates and higher life expectancy are transforming the shape of the EU-28’s age pyramid; probably the most important change will be the marked transition towards a much older population structure, a development which is already apparent in several EU Member States [3].

The population of the EU-28 on 1 January 2016 was estimated at 510.3 million. Young people (0 to 14 years old) made up 15.6 % of the EU-28’s population, while persons considered to be of working age (15 to 64 years old) accounted for 65.3 % of the population. Older people (aged 65 or over) had a 19.2 % share (an increase of 0.3 % compared to the previous year and an increase of 2.4 % compared to 10 years earlier) [4].

**Figure 1:**
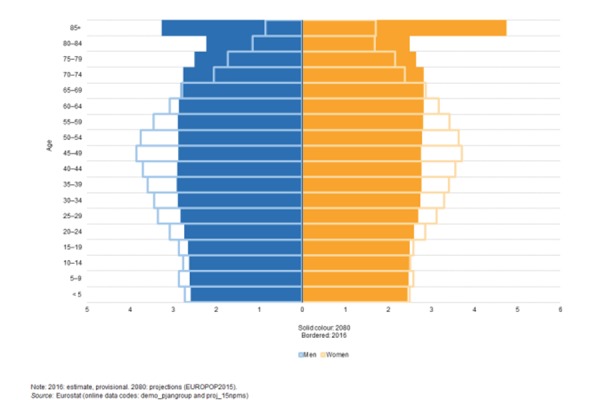
Population pyramide EU-28, 2016 and 2080 (% of the total population)

According to projections [4], the overall size of the population is projected to be slightly larger by 2070 than in 2016. The EU-28 population is projected to increase by about 3.5% between 2016 (511 million) and 2040 (at 528 million) when it will peak, to then remain stable until 2050 and to thereafter decline to 520 million in 2070 (see Figure 1). While the total EU-28 population will increase by 1.8% over 2016–70, there are wide differences in population trends across the Member States, with the population increasing in half of the EU countries and falling in the other half.

The demographic old-age dependency ratio (people aged 65 or above relative to those aged 15–64) is projected [3] to increase significantly in the EU as a whole in the coming decades. Being about 25% in 2010, it has risen to 29.6% in 2016 and is projected to rise further, in particular up to 2050, and eventually reach 51.2% in 2070.

This implies that the EU would move from four working-age people for every person aged over 65 years in 2010 to around two working-age people over the projection horizon. As a result, the proportion of people at working age in the EU-28 is shrinking while the relative number of those retired is expanding [3].

The share of older people in the total population will increase significantly in the coming decades, as a greater proportion of the post-war baby-boom generation reaches retirement. This will, in turn, lead to an increased burden on those at working age to provide for the health and social expenditure required by the ageing population for a range of related services.

Health care services represent a high and increasing share of government spending and total age-related expenditure. Furthermore, the ageing of the EU population may entail additional government expenditure. This makes public spending on health care an integral part of the debates on the long-term sustainability of public finances.

The projection [3] for those aged 80 years and more will almost triple by 2060. This trend will cause an increase of social expenses in forms of pensions, healthcare and institutional or private care. Under this scenario, public spending on the older people will be a major problem in upcoming years.

This demographic change will have considerable consequences for the EU public finances. Based on current policies, it is estimated that **‘exclusively’ age-related (pensions, health, and long-term care) public expenditure will increase by 4.1 percentage points of GDP between 2010 and 2060, from 25% to 29%**. Only expenditure on pensions is expected to increase from 11.3% to nearly 13% of GDP by 2060. However, there are significant differences between countries, depending largely on the progress made by each country in the reform of the pension system, which confirms the need for policy action to meet the challenges of an ageing population.

## II. METHODOLOGY

SHAFE aims to facilitate the creation of healthy and friendly environments for all ages through the use of new technologies. In more concrete terms, it is intended to highlight the importance of People and Places in the creation of digital solutions for eHealth and mHealth, with better quality, but still accessible to all. The main aim is to value the Person as a central element of the whole process of digitisation.

This Thematic Network has created a high-level political alignment of different networks and initiatives for age-related themes. It is aligned with the EU Health Priorities [5] in creating synergies that will increase quality, innovation and sustainability for the implementation of better health and care systems, economic growth and sustainable health, in line also with the objectives of the Blueprint and Communication on Digital Transformation of Health and Care [6][7].

Moving age-related topics to the big umbrella themes of the Health and Digital Single Market is a vital process to pursue the societal scope of a Europe prepared to provide quality of life and well-being throughout the whole life cycle. The revitalisation of Active and Healthy Ageing initiatives (preparing post-2020) will imply high-level crossover discussions between different groups, networks, Directorates of the European Commission (DGs), European Innovation Partnerships (EIPs) and even international organisations, understanding the symbiotic interdependence of these subjects towards a Healthy and Competitive Europe. This Thematic Network intends to create a high-level policy alignment of all these networks and initiatives towards Health in Ageing subjects.

The specific aim of SHAFE is to enhance the two main aspects of Age-Friendly Environments – Places and People – in the creation of eHealth and mHealth solutions - especially focused on quality and costs.

On eHealth, a special emphasis is given to its current state of the art in e-support of smart homes to people who suffer from chronic diseases and impairments - e-support like robotics, smart living environments and smart communication with formal and informal care providers. These smart environments need to align physical and technological development with the building industry in terms of policy and funding, in order to make smart homes available, affordable, and large-scaled throughout Europe. This broad adoption may be the keystone to more efficient health care systems that add better quality for less investment.

On mHealth the focus is on understanding and bridging the main gaps between technological development and real user needs and expectations, proposing policy measures that favour and enhance a real market entrance of new solutions, hoping to decrease inequalities in the access to health services.

The partnership of the Thematic Network is developed in a quadruple layer-scheme, with the intention to implement a Europe-wide network of stakeholders that actually provide inputs to the Joint Statement framing paper and call to action:

### 1. Coordinators

Cáritas Coimbra and AFEdemy develop the overall strategy of the Thematic Network, coordinate the partnership contributions, tasks, and roles, provide the dissemination materials and external communications and represent SHAFE in events and by the European Commission. They also developed the main guidelines of the framing paper and call to action and were responsible for the final edition of the document presented as Joint Statement.

### 2. Main partners

The main partners are the European organisations and networks that supported the Thematic Network official proposal [2]:

- European Innovation Partnership on Active and Healthy Ageing (EIP-AHA)^3^
- European Innovation Partnership on Smart Cities and Communities (EIP-SCC)^4^
- Reference Sites Collaborative Network^5^?
- European Covenant on Demographic Change^6^?
- Eurocities^7^?
- European Health Telematics Association (EHTEL)^8^
- ECHAlliance^9^?
- European Construction, Built Environment and Energy Efficient Building Technology Platform (ECTP)^10^
- European Centre for Social Welfare Policy and Research^11^

### 3. Associated partners

The associated partners are all organisations and networks that cooperate with the coordinators by delivering work, suggestions and comments on the Framing Paper and Joint Statement. There were 109 registered partners on November 5^th^, 2018.^12^

### 4. Endorsing partners

The endorsing partners are all networks or organisations that subscribe to the final version of the Joint Statement presented to the European Commission on November 12^th^, 2018.

SHAFE’s main objectives are the following [2]:

- Produce a Joint Statement in 2018 that summarises a common position on Smart Healthy Age-Friendly Environments, priorities for policy making and recommendations beyond 2020, aiming at a White Paper in 2019/2020;
- Provide a forum to exchange policy priorities and technical expertise on Age-Friendly Environments and eHealth and mHealth solutions;
- Inform the European Commission and the Member States on knowledge and expertise available in the stakeholder community about challenges, solutions, and best practices on Age-Friendly Environments and eHealth/mHealth;
- Bring better local practices already implemented by local and regional authorities that have been identified in the EIP-AHA for twinning or scaling-up and collect lessons learned towards policy drawing;
- Promote common principles as person-centered interventions, protection of personal data, standardisation, interoperability, data-enabled research, personalised medicine, and quadruple helix.

As a departing point to the research activities, four questions were defined. The answers to these questions shaped SHAFE’s outcomes:

1. How to enhance Places and People in the use and installation of eHealth and mHealth solutions, with special focus on quality and costs?
2. What is the current state of the art in Europe in terms of e-support at home to people with chronic disease and/or impairments?
3. How to align technological development with the building industry for smart environments in terms of policy and funding, enhancing a more efficient health care system that adds better quality for less investment?
4. How to bridge the main gaps between technological development and the user’s needs and expectations?

The research activities were executed, and the first results were available on middle-June 2018 [8].

The research was executed by performing:

- Desk research - using dedicated search terms in databases such as Google Scholar, PubMed, Cochrane, Scopus, WorldCat, PiCarta, Web of Science, ACM Digital Library, NARCIS, OATD, DOAJ, BASE, CORE, Paperity, AAL-database, CORDIS, and Innoradar.eu. It included search in grey literature in EU countries, using search terms in own languages by associated partners.
- Survey published online in the EU Survey website and broadly disseminated through the networks of the Coordinators and Main partners.
- Interviews with several opinion leaders on the topics related to eHealth, mHealth solutions, active ageing, Age-Friendly Environments, chronic diseases, and impairments, living independently, with the help from associated partners.

Besides research, several activities of discussions, comment and support on the Joint Statement and research results were performed through events and dissemination activities, namely:

- EIP-AHA Action Group meeting in Manchester (2–3 July 2018).
- AAL Forum/Silver Week Bilbao 2018 (24–26 September) – Workshop 12.
- Online consultation through SHAFE’s webpage^13^.?
- Thematic Network webinars on June 19^th^ and October 9^th^, 2018.

## III. RESULTS

The desk research, survey and interviews resulted in answers on above mentioned questions as laid down in the Framing Paper of the network [8].

1. To enhance People in the use and installation of eHealth and mHealth solutions, it is necessary to bridge the digital gap that is still present in European society. Lifelong learning and co-creation are valuable means hereto. Construction plans for new buildings and houses should already take into consideration guidelines and standards that allow for adequate and appropriate conditions for current and upcoming ICT demands.
2. Easy and accessible overviews about the current state of the art regarding e-support at home were mostly missing in appropriate databases of funded projects. There is the need for efficient accessibility options of all the data collected over the years in such a way that it may be re-used and integrated into future research, policy-making and societal progress.
3. Construction industries and technology companies need to be aligned to jointly develop and reconstruct new and existing houses, public buildings, healthcare facilities, transportation facilities, and outdoor spaces. To achieve the required and more integral approach it is needed to encourage partnerships at a local and regional level to get a better knowledge exchange between these, namely in shared guidelines and standards.
4. To bridge the main gaps between technological development and user’s needs and expectations the only solution is to involve users from the beginning when planning, designing, and constructing new or adapting living environments and technology, guaranteeing that the real user’s needs and challenges are addressed.

Following the outcomes of the desk-research, interviews, and survey, the following five main areas were identified [8]:

- Integrative approach?
- Governance and coordination?
- Funding, economic, and business models?
- Learning and knowledge management?

Communication, people, and societal challenges Departing from the outcomes collected for each of these areas, SHAFE identified five significant recommendations as the “*chapeau*” for the Joint Statement. In consultation and discussion with the main and associated partners and with the inputs collected at the AAL Forum (September 25^th^, workshop 12) and during the second Thematic Network webinar (October 2^nd^), the recommendations were further elaborated, detailed in more targeted actions and refined.

## IV. DISCUSSION

The recommendations issued in the Joint Statement may be read in the official document [9] published in SHAFE’s official website and may be summarized as follows:

### Call 1 | Create a shared European vision on Smart Healthy Age-Friendly Environments

Our first recommendation is to develop a shared European vision on Smart Healthy Age-Friendly Environments (SHAFE) that may be used as an inspiring sketch to be used at local and regional level everywhere in Europe.

We urge the European Commission and the Member States to cooperate with the partners of SHAFE, the Steering Group and Local and Regional Authorities to create the conditions to have this high-level agreement among relevant actors in Europe, recognizing that Smart Healthy Age-Friendly Environments can benefit the whole society and involve international agencies, public authorities, as well as civil society and NGOs.

### Call 2 | Promote cross-sectoral cooperation

To achieve a better integrative collaboration, we recommend the creation of national and international interdisciplinary policy and societal working groups or ecosystems at all levels that are responsible for developing joint policies of the implementation of Smart Healthy Age-Friendly Environments. The European common vision can be an inspiration.

Secondly, equal and easy access to information and knowledge is crucial to build-up equal starting-points for every stakeholder and thus to further foster successful cross-sectoral cooperation.

To achieve further cross-sectoral cooperation, EU policy shall be aligned with the Member States on the integration of public welfare with health and social care system and also with private providers.

### Call 3 | Fund the implementation of Smart Healthy Age-Friendly Environments

The partners recognize the need for a big push to implementation. This can be achieved by fostering positive discrimination in budget and public funding to organisations that work on Smart Healthy Age-Friendly Environments. A shift to funding of upscaling of already existing, well-performing examples would better enhance the roll out across Europe.

It is very important that European and national funding schemes are aligned so that innovation coming from successful European projects can be integrated into national frameworks and scaled-up through Europe. New ways of implementing this specific ”dialogue” between different funding programmes must be a priority.

Also, very important is to create funding frameworks that integrate different elements on shafe, such as health, social care, ICT and built environments in the same mission and calls, allowing effective implementation of long-term sustainable solutions.

Besides funding, also public procurement is an essential element to give a boost to large-scale implementation.

### Call 4 | Invest in research that derives from societal needs and challenges and use knowledge to prediction and prevention

The better way to guarantee sustainability is to invest in prevention through the life course and predict the upcoming challenges and changes with enough time to address them with the minimum resources.

To achieve more impacts regarding societal needs and challenges, we recommend increasing the demand on the social and economic impact of projects which may constitute an opportunity and area of future investment for researchers.

Research is essential to predict and prevent what may be the biggest challenges of societies in the years to come.

### Call 5 | Guarantee the empowerment of citizens and the promotion of people-centered policies and measures

Sustainable development needs to start earlier: having a lifelong approach implies that it starts even before birth. Citizens need to be involved from the start of each development that concerns their living environments and their health and care.

Moving from treating diseases to prevention and well-being is critical. Mindsets need to change, and education is the key. Promotion of healthy lifestyles and well-being makes people autonomous through education.

It is also necessary that citizens get the opportunity to initiate improvements in their living environments themselves. This does not intend to pass the responsibility for the citizens but instead to promote a culture of shared responsibility between authorities and citizens – all working together towards the common good.

## V. CONCLUSION

Cáritas Coimbra and AFEdemy will maintain to foster the network towards the implementation of the recommendations at all levels – European, national, regional and local. The work developed is not only requested at a high policy level to the EC and the Member States, but all SHAFE partner organisations are committed to working also on their implementation as active stakeholders.

Cáritas Coimbra and AFEdemy also intend to take the Joint Statement a step further into a White Paper during 2019/2020 and launched the works officially at ICT 2018, the EC Conference that was held from 4–6th December in Vienna, Austria.

This was the beginning of a public process on the construction of the White Paper on SHAFE, open to discussion and engagement around Europe. Connections between the different networks created throughout the discussion will drive to breaking silos and defining common strategies and initiatives.

## Footnotes

3 https://ec.europa.eu/eip/ageing/home_en

4 https://eu-smartcities.eu/

5 http://www.rscn.eu/

6 https://www.agefriendlyeurope.org/

7 http://www.eurocities.eu/

8 https://www.ehtel.eu/

9 https://echalliance.com/

10 http://www.ectp.org/

11 https://www.euro.centre.org/

12 https://en.caritascoimbra.pt/shafe/associated-partners/

13 https://en.caritascoimbra.pt/shafe/
